# Association between Serum Ferritin and Osteocalcin as a Potential Mechanism Explaining the Iron-Induced Insulin Resistance

**DOI:** 10.1371/journal.pone.0076433

**Published:** 2013-10-22

**Authors:** Martí Juanola-Falgarona, José Cándido-Fernández, Jordi Salas-Salvadó, Miguel A. Martínez-González, Ramón Estruch, Miquel Fiol, Victoria Arija-Val, Mònica Bulló, for the PREDIMED Study Investigators

**Affiliations:** 1 Human Nutrition Unit and Preventive Medicine Unit, Faculty of Medicine and Health Sciences, IISPV, Universitat Rovira i Virgili, Reus, Spain; 2 CIBERobn Physiopathology of Obesity and Nutrition, Institute of Health Carlos III (ISCIII), Madrid, Spain; 3 Department of Preventive Medicine and Public Health, University of Navarra, Pamplona, Spain; 4 Department of Internal Medicine, Institut d'Investigacions Biomèdiques August Pi Sunyer, Barcelona, Spain; 5 University Institute for Health Sciences Investigation, Palma de Mallorca, Spain; Omaha Veterans Affairs Medical Center, United States of America

## Abstract

**Background:**

Increased iron stores are associated with increased risk of type 2 diabetes, however, the mechanisms underlying these associations are poorly understood. Because a reduction of circulating osteocalcin levels after iron overload have been demonstrated in cell cultures, and osteocalcin is related to glucose and insulin metabolism, the iron-induced osteocalcin reductions could contribute to explain the role of iron metabolism in the development of type 2 diabetes mellitus.

**Objective:**

To analyzed the associations between serum total and uncarboxylated osteocalcin and adiponectin concentrations with serum ferritin and soluble transferrin receptor (sTfR) in elderly subjects.

**Design:**

We evaluated a total of 423 subjects from the PREDIMED cohort in a population-based cross-sectional analysis. Extensive clinical, nutritional and laboratory measurements, including total and uncarboxylated osteocalcin, adiponectin, ferritin and sTfR were recorded.

**Results:**

Serum ferritin was positively correlated with increased glucose and insulin circulating levels but also with HOMA-IR, and was inversely associated with total osteocalcin and adiponectin. A regression analysis revealed that serum ferritin and transferrin receptor levels were significantly associated with a decrease in total and uncarboxylated osteocalcin. Serum sTfR levels were associated with lower uncarboxylated osteocalcin levels in the whole-study subjects and remained significant only in the IFG (impaired fasting glucose) individuals.

**Conclusions:**

We described, for the first time, an inverse association between serum ferritin and sTfR with osteocalcin and extend previous results on adiponectin, thus supporting that factors related to iron metabolism could contribute to the insulin resistance and the development of type 2 diabetes mellitus.

**Trial Registration:**

Controlled-Trials.com ISRCTN35739639 <ISRCTN35739639>.

## Introduction

Iron is an essential mineral for humans although is potentially hazardous in excess amounts. Excessive iron stores in patients with hereditary hemochromatosis (HH) have been causally related with the development of type 2 diabetes mellitus (T2DM) [1]. However, moderately increased iron stores are also associated with hyperglycemia and hyperinsulinemia, or an increased risk of type 2 diabetes mellitus in apparently healthy subjects [2]–[5]. These findings have been confirmed in a recent meta-analysis of five prospective epidemiologic studies, giving a pooled RR of 1.63 for type 2 diabetes mellitus in subjects with the highest levels of ferritin [6]. Additionally, in a nested case-control study, increased soluble transferrin receptor (sTfR) levels were associated with increased T2DM risk (OR 2.26 [1.37–4.01] [7]. Increased iron stores have also been associated with gestational diabetes [8], prediabetes [9], central adiposity [10], metabolic syndrome [11], cardiovascular disease [12] and osteopenia or osteoporosis [13], [14].

Possible mediators linking iron stores and diabetes are still poorly understood. Serum ferritin levels, the commonly used marker for total body iron stores, has been associated with insulin resistance measured by homeostasis model assessment (HOMA IR) or hyperinsulinemic euglycemic clamp [15], [16] but not with pancreatic beta-cell function in humans [3], whereas in obese mouse, dietary iron restriction protects from loss of beta cell function [17]. Iron deposition in the muscle decreases glucose uptake due to muscle damage [18] and it has additionally been suggested that iron deposition in pancreatic β-cells impairs insulin secretion in more advanced states of iron overload [19]. Despite that, the effect of iron depots on other insulin-related tissues such as adipose or bone tissues is far from clear. Recent studies conducted in animals or in humans have demonstrated a direct and causal effect of iron stores in circulating levels of adiponectin, independently of other peripheral markers of inflammation [20], [21], thus explaining the attenuated association between ferritin and incident type 2 diabetes mellitus observed after adjustment for circulating adiponectin levels [5]. Additionally, a dose-response decreased expression of genes related to the osteoblast phenotype, including osteocalcin, after iron overload have been demonstrated in cell cultures [22]–[24]. Thus, because osteocalcin (OC) has been related to a decrease in fasting glucose concentrations and to an increase of pancreatic beta-cell proliferation, insulin secretion and sensitivity [25], the iron-induced osteocalcin reductions could contribute to explain the role of iron overload in the development type 2 diabetes mellitus.

To our best knowledge, there are no studies demonstrating a direct association between markers of iron metabolism and osteocalcin concentrations in humans. We therefore conducted the present study to evaluate possible associations between serum total and uncarboxylated osteocalcin concentrations with serum ferritin and (sTfR) in elderly subjects at high cardiovascular risk.

## Methods

### Study design and population

For the present analysis, non-diabetic participants from three Spanish centers (Reus-Tarragona, Navarra and Barcelona-Clinic) within the framework of the PREDIMED study were randomly selected. The PREDIMED study is a multicenter, randomized clinical trial conducted in Spain to assess the effects of the Mediterranean diet (MedDiet) on the primary prevention of cardiovascular disease. The design of the PREDIMED trial (http://www.controlled-trials.com/ISRCTN35739639) has been reported elsewhere [26], [27], and it is available at http://www.predimed.org and www.predimed.es. Subjects were men aged 55 to 80 years and women aged 60 to 80 years without prior cardiovascular disease (CVD) at baseline but with at least three cardiovascular risk factors namely: smoking, hypertension, dyslipidemia, overweight (Body mass index (BMI) ≥25 kg/m^2^), and a family history of early-onset coronary heart disease (before age 55 years in men or before age 65 years in women) in first-degree relatives [27]. The exclusion criteria for the PREDIMED study were any severe chronic illness, alcohol or drug addiction, history of food allergy to olive oil or nuts, or a low predicted likelihood of changing dietary habits according to Prochaska and DiClemente's stages-of-change model [28].

We performed the current cross-sectional analyses at baseline evaluation of a subsample of 455 subjects of this cohort, after excluding 9 participants with very low values of serum ferritin who were clearly outside the normality levels according to the cut-off of our central laboratory to exclude individuals with ferropenia (<10 ng/dL in women and <20 ng/dL in men). We also excluded other 23 subjects with missing data on plasma glucose, insulin or some important covariates for the analysis.

The PREDIMED study protocol was approved by the institutional review boards of all the centres involved, and all subjects agreed to participate in the study and gave their written informed consent.

### Measurements

Medical information was collected on subjects' medical record of a 47-item questionnaire about education, lifestyle, history of illnesses and medication use. A validated 137-item food frequency questionnaire (FFQ) was administered [29] and dietary energy intake was calculated from Spanish food composition tables [30]. Trained personnel measured baseline weight, height and waist circumference as previously reported, as well as blood pressure in triplicate with a validated semiautomatic oscillometer (Omron HEM-705CP, Hoofddorp, Netherlands). Enegy expenditure in leisure-time physical activity (LTPA) was estimated using the validated Spanish version of the Minnesota Questionnaire.

Blood samples were collected from all participants after an overnight fast and were immediately processed, coded, and shipped to a central laboratory. Serum levels of fasting glucose, total cholesterol, HDL-cholesterol and triglycerides were measured by standard enzymatic automated methods. LDL-cholesterol concentrations were calculated using Friedewald's equation in those patients whose triglyceride levels were <400 mg/dL. Plasma fasting insulin concentrations were measured by an ELISA kit for human insulin (Millipore, St. Charles, Missouri, USA). Insulin resistance was estimated by the HOMA method using the following equation [31]: HOMA-IR  =  [fasting insulin (μIU/mL) x fasting glucose (mmol/L)]/22.5. Serum total and uncarboxylated osteocalcin levels were measured by electrochemiluminescence immunoassay (N-mid osteocalcin, Roche, Indianapolis, IN); the intra- and inter-assay coefficients of variation were <3.6% and <6.6% respectively). Altered beta-cell function was estimated using HOMA-BCF (homeostasis model assessment-beta cell) as previously described [32]. Serum ferritin and soluble transferrin receptor were measured by a particle-enhanced immunoturbidimetric assay using the Hitachi analyzer and sTfR:ferritin ratio was calculated. C-reactive protein (CRP) concentrations were measured via a highly sensitive immunoassay (Helica Biosystems Inc, Santa Ana, CA). Adiponectin levels were measured using an enzymatic immunoassay (Millipore, St. Charles, Missouri, USA). Plasma oxidized LDL (oxLDL) concentrations were also measured by a commercial ELISA (Mercodia Oxidized LDL ELISA, Uppsala, Sweden).

### Statistical analysis

Variables with skewed distribution according to the Kolmogorov–Smirnov tests were log_e_-transformed before analysis. Means (SE), percentages (%) or median (IQR) were used for descriptive purposes. Whether the associations differed between sexes were tested by adding the interaction terms sex*ferritin and sex*sTfR to the fully adjusted models. Because no interaction was found, all results are presented for the whole population studied. Although no interaction was observed between markers of iron status and glucose metabolism (IFG*ferritin and IFG*sTfR) it is physiologically plausible that impaired fasting glucose (IFG) (considered when fasting glucose levels were higher than 100 mg/dL) could affect the associations between ferritin and sTfR with total and uncarboxylated osteocalcin or adiponectina [33]. For this reason, results are also presented stratified according to glucose metabolism for two different groups: for subjects with normal glucose metabolism (NGM) and for those with IFG. General characteristics of the study subjects were compared between both sexes using ANOVA. Associations between markers of iron metabolism (ferritin, transferrin receptor and the ratio sTfR:ferritin) and metabolic or inflammatory variables were examined using Spearman's correlations. To examine the relationship between markers of iron metabolism with total or uncarboxylated osteocalcin, linear regression models were fitted including sex, age, BMI, smoking status, total energy intake, energy expenditure in leisure-time physical activity, fasting plasma glucose and insulin, and markers of inflammation or oxidation (adiponectin, CRP, oxLDL) as potentially confounding variables in the fully adjusted model. In addition, both regressions analyses with either serum ferritin or sTfR as outcomes were mutually adjusted for each other to account for negative confounding because they are both independently associated with insulin resistance despite their negative reciprocal correlation [16], [34]. For log_e_-transformed outcome variables (i.e. total and uncarboxylated osteocalcin) regression coefficients were converted into percentages of relative change in the original variable (percentage decrease in total or uncarboxylated osteocalcin per 50 ng/mL increase in ferritin or per 1 ng/mL of increased sTfR). All analyses were performed using the SPSS 20.0 software (SPSS Inc, Chicago, IL).

## Results

General characteristics of the study subjects are reported in **Table 1**. Total and uncarboxylated osteocalcin and adiponectin were significantly lower in subjects with impaired fasting glucose. A tendency to higher ferritin concentrations and a lower ratio of sTfR:ferritin were observed in individuals with impaired fasting glucose in comparison to those with normal glucose metabolism as expected, although these differences didn't reach statistical significance.

**Table 1:** Baseline characteristics of the study subjects.

	Total subjects	NGM	IFG	P
**N**	423	250	173	
**Men/women**	202/221	111/139	91/82	0.097
**Age (years)**	66.3±0.3	66.3±0.4	66.2±0.4	0.902
**BMI (kg/m^2^)**	29.6±0.1	29.4±0.2	29.8±0.2	0.161
**Waist circumference (cm)**	99.00±0.45	97.94±0.60	100.44±0.70	0.007
**Total energy intake (Kcal/day)**	2377.75±28.78	2368.37±37.20	2391.21±45.53	0.707
**Physical activity (METs-min/day)**	273.4±11.6	272.4±15.3	274.9±17.9	0.916
**Smoking habit (yes/no)**	96/327	59/191	37/136	0.757
**Fasting glucose (mg/dL)**	97.00 (89.01, 109.01)	90.00 (85.00, 95.29)	111.15 (105.00, 122.75)	<0.001
**Total cholesterol (mg/dL)**	223.21±1.84	223.07±2.22	223.41±3.17	0.929
**HDL cholesterol (mg/dL)**	55.29±0.67	56.67±0.95	53.30±0.89	0.014
**LDL cholesterol (mg/dL)**	139.61±1.57	139.59±2.02	139.63±2.51	0.990
**Triglyceride level (mg/dL)**	141.82±4.27	133.73±4.51	153.57±8.12	0.022
**Fasting plasma insulin (mU/mL)**	4.61 (3.12, 7.01)	4.23 (3.06,6.48)	5.04 (3.332,7.61)	0.037
**HOMA-IR**	1.35±0.04	1.14±0.04	1.68±0.07	<0.001
**HOMA-BCF %**	63.12±2.88	77.37±4.40	41.93±1.87	<0.001
**Total osteocalcin (ng/mL)**	8.00 (6.17,10.75)	8.50 (6.36, 11.52)	7.44 (5.78,9.64)	0.005
**Uncarboxylated osteocalcin (ng/mL)**	4.15 (2.38,6.06)	4.58 (2.59,6.80)	3.56 (2.15,5.46)	0.003
**Ratio ucOC/OC**	0.58±0.02	0.62±0.03	0.53±0.03	0.077
**Adiponectin (ng/mL)**	8.30 (5.29,13.22)	9.15 (6.10,14.23)	6.98 (4.68,11.54)	0.001
**C-Reactive Protein (ng/mL)**	1.32 (0.26,3.58)	1.24 (0.22,3.17)	1.43 (0.31,3.90)	0.612
**oxLDL (mU/L)**	59.17±1.26	60.58±1.62	57.11±2.01	0.150
**Ferritin (ng/mL)**	126.20 (67.98, 212.47)	118.85 (66.64, 204.75)	132.70 (75.38, 230.91)	0.104
**Transferrin receptor (mg/L)**	1.24 (1.07,1.42)	1.25 (1.09,1.43)	1.22 (1.07,1.41)	0.242
**Ratio Transferrin/ferritin**	0.018±0.0012	0.019±0.0018	0.015±0.027	0.155

Data expressed as mean ± SE, mean (IQR). P* are differences between normal glucose metabolism (NGM) and impaired fasting glucose (IFG) groups.

**Figure 1:**
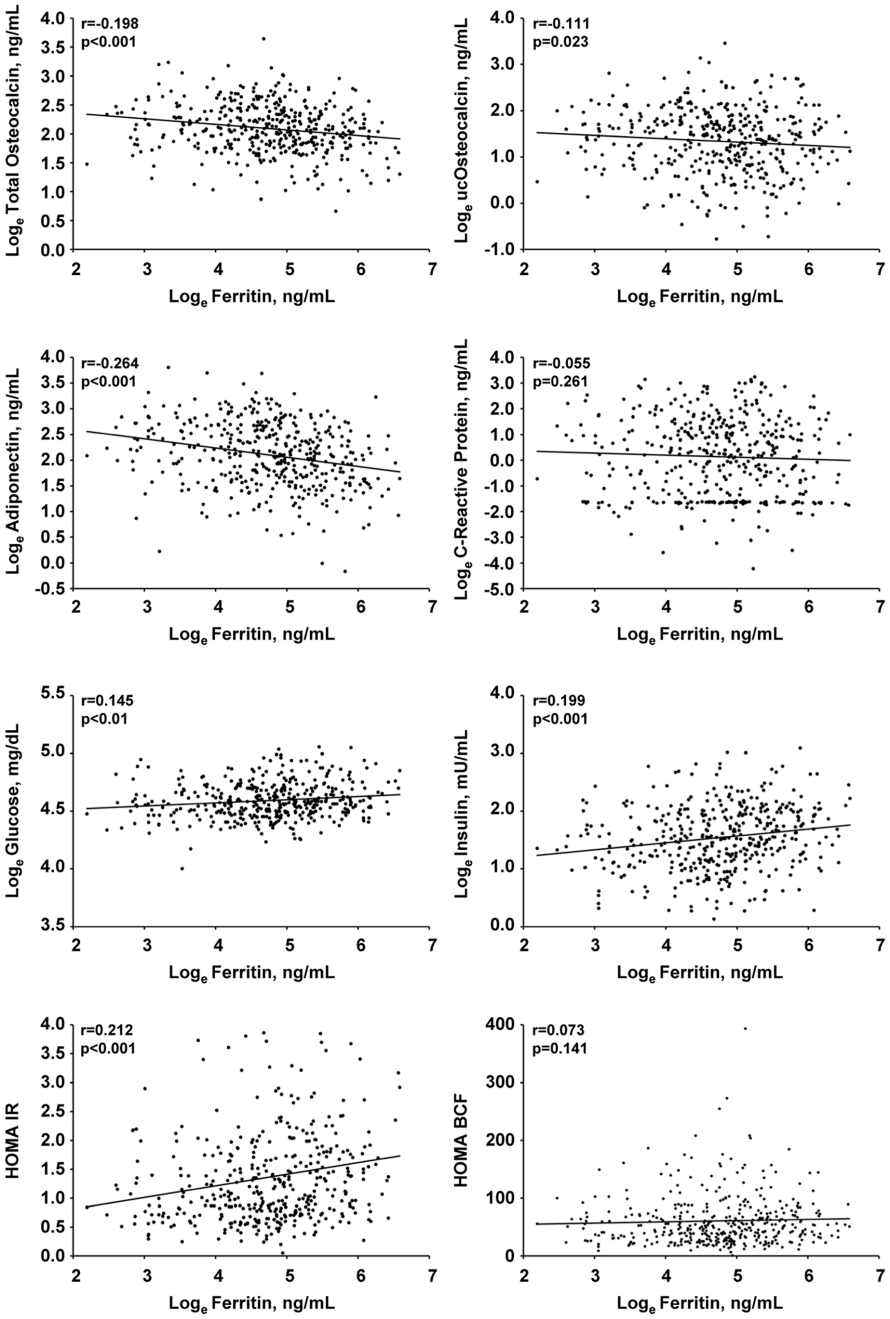
Correlations between serum ferritin levels and selected biochemical parameters. Correlation coefficients are based on log_e_-transformed values of markers except HOMA-IR and HOMA-BCF.

In the whole-study population, serum ferritin was positively correlated with increased glucose and insulin circulating levels but also with HOMA-IR, and was inversely related with total and uncarboxylated osteocalcin and adiponectin but not with HOMA-BCF (**Figure 1**). Soluble transferrin receptor concentrations were positively related with plasma oxLDL levels (r = 0.134, p = 0.006) and non- related with plasma glucose (r = −0.078, p = 0.113, insulin (r = 0.003, p = 0.946), HOMAIR (r = −0.032, p = 0.522) and HOMA-BCF (r = 0.014, p = 0.771). A non-significant correlation was observed between sTfR and ucOC (r = −0.088, p = 0.071), and between sTfR:ferritin and glucose, insulin and HOMA-IR (r = −0.154, r = −0.177, r = −0.194 p<0.001 for all).

Total OC and adiponectin were negative associated with HOMA-IR (−0.193 and −0.338, p<0.001 respectively). In addition, ferritin and sTfR serum levels were inversely correlated (r = −0.250, p<0.001). In a linear regression model, serum ferritin and transferrin receptor concentrations were significantly associated with a decrease in total OC (p = 0.004) per 50 ng/mL of increased ferritin, and a decrease in uncarboxylated osteocalcin (ucOC) (p = 0.038) per 1 ng/mL of increased sTfR even after adjustment for sex, age, BMI, smoking status, total dietary energy intake, physical activity, fasting plasma glucose, and insulin, and peripheral markers of inflammation and oxidation (adiponectin, CRP, oxLDL), and sTfR or serum ferritin (for models with ferritin or TfR as the outcome, respectively). These associations were apparent both in normoglycemic and in subjects with impaired fasting glucose, although in the last case the association between ferritin and total OC was not statistical significance (p = 0.080). Serum sTfR levels were associated with lower ucOC levels in the whole-study subjects and remained significant only in the IGM individuals (**Table 2**). In contrast, sTfR:ferritin ratio was not significantly associated nor with total OC neither with ucOC.

**Table 2:** Association between markers of iron metabolism and total or uncarboxylated osteocalcin serum levels.

	For each 50ng/mL of increased ferritin						For each 1ng/mL of increased sTfR					For each 0.1 ng/mL of increased ratio sTfRferritin		
*Outcome*	Regression coefficient		95% CI		P-value		Regression coefficient	95% CI		P-value		Regression coefficient	95% CI	P-value
***In the whole population***														
**Total OC (ng/mL) (% change)**														
Crude	−3.82		−5.25, −2.27		<0.001		−8.05	−19.42, 4.91		0.211		9.85	−6.48, 29.04	0.250
Model 1	−2.76		−4.30, −1.09		0.001		−6.76	−17.96, 6.07		0.286		5.02	−10.41, 23.24	0.543
Model 2	−2.46		−4.01, −0.79		0.004		−12.97	−23.73, −0.79		0.038		0.80	−13.92, 18.05	0.921
**Uncarboxylated OC (ng/mL) (% change)**														
Crude	−1.98		−4.49, 0.70		0.140		−17.05	−33.16, 2.83		0.088		7.03	−17.79, 39.37	0.612
Model 1	−0.29		−2.95, 2.53		0.859		−17.55	−33.30, 2.02		0.075		−2.95	−25.54, 26.49	0.825
Model 2	−0.39		−3.24, 2.42		0.765		−22.11	−37.76, −2.56		0.029		−5.25	−27.45, 23.86	0.694
***In the NGM***														
**Total OC (ng/mL) (% change)**														
Crude	−4.11		−6.26, −1.98		<0.001		−14.27	−27.31, 1.00		0.066		0.60	−16.80, 21.65	0.951
Model 1	−2.95		−5.25, −0.59		0.013		−10.05	−23.58, 5.86		0.200		0.002	−17.30, 20.92	0.998
Model 2	−2.95		−5.06, −0.69		0.010		−16.13	−29.03, −0.98		0.038		−4.87	−21.25, 14.91	0.603
**Uncarboxylated OC (ng/mL) (% change)**														
Crude	−0.99		−4.78, 2.94		0.620		−16.97	−37.12, 9.52		0.186		−1.39	−28.46, 35.79	0.929
Model 1	1.00		−3.05, 5.01		0.644		−14.61	−35.07, 12.29		0.257		−9.87	−34.62, 24.11	0.521
Model 2	0.40		−3.72, 4.81		0.815		−16.13	−37.37, 12.29		0.236		−10.05	−35.20, 24.85	0.542
***In the IFG***														
**Total OC (ng/mL) (% change)**														
**Crude**		−3.14		−5.16, −0.98	0.005		0.90		−18.86, 25.35		0.939	32.04	−2.37, 78.42	0.071
**Model 1**		−2.37		−4.59, −0.19	0.036		−1.29		−20.30, 22.14		0.901	15.83	−14.78, 57.45	0.345
**Model 2**		−2.17		−4.49, 0.30	0.080		−7.03		−25.91, 16.64		0.526	14.50	−15.88, 55.89	0.386
**Uncarboxylated OC (ng/mL) (% change)**														
**Crude**		−2.27		−5.63, 1.10		0.183	−22.81		−44.95,8.22		0.132	20.44	−25.09, 93.67	0.441
**Model 1**		−1.39		−4.87, 2.32		0.468	−25.53		−48.31, 1.51		0.061	3.97	−36.36, 69.89	0.875
**Model 2**		−2.07		−5.82, 1.91		0.308	−34.29		−54.38, −5.35		0.024	3.35	−37.37, 70.57	0.897

β values are unstandardized regression coefficients and represents the change in total and uncarboxylated osteocalcin according to increases in ferritin, soluble transferrin receptor and the ratio of sTfR/ferritin. Model 1: adjusted for sex, age and BMI, smoking status, total energy intake and energy expenditure in leisure-time physical activity. Model 2: additionally adjusted for fasting plasma glucose, insulin and markers of inflammation and oxidative stress (adiponectin, C-reactive protein, oxidized LDL) and ferritin and transferrin receptor adjusted for each other).

## Discussion

The present study confirmed, for the first time, that both serum ferritin and soluble TfR levels, as markers of iron metabolism, are independently and inversely associated with total and uncarboxylated osteocalcin. Because osteocalcin has been related not only with bone metabolism but also with insulin resistance and sensitivity, our results would help to explain one of the possible mechanisms relating iron metabolism, insulin resistance and risk of type 2 diabetes mellitus. We also confirm and extend previous results showing the inverse association between serum ferritin and adiponectin concentrations as a potential mechanism linking iron stores with insulin resistance [21], [35].

Serum OC levels were used to evaluate bone metabolism because it has been considered a better sensitive marker of bone formation than serum alkaline phosphatase. However, increasing data supporting extra-skeletal roles of OC have emerged, being widely accepted its hormonal effect on energy metabolism, angiogenesis or insulin metabolism [36]. In this sense, mice lacking osteocalcin show lower beta-cell proliferation, glucose intolerance, and insulin resistance than wild-type mice [15]. Additionally, osteocalcin knockout mice have reduced levels of serum adiponectin, which suggests a potential role for osteocalcin in insulin sensitivity and secretion. In a previous study, our group demonstrated, for the first time, that increased total or uncarboxylated OC serum concentrations were directly associated with HOMA-BCF and inversely with insulin resistance determined by HOMA-IR [25].

Iron overload can damage several important organs such as liver, pancreas and heart. Many lines of evidence indicated that iron overload affects bone tissue causing both osteopenia and osteoporosis [37]. Addition of iron to a culture of human osteoblasts decreased osteocalcin concentrations dose-responsively. This has been attributed to the ferroxidase activity of ferritin rather than its iron sequestering capacity [24]. Furthermore, iron exposure on human osteoblast-like cells reduced the expression of genes involved in bone matrix formation or osteoblast differentiation such as COL1AI, Runx2 or osteocalcin [23], [38]. The observed inverse associations of ferritin with total and uncarboxylated osteocalcin observed in our study, independently of other inflammatory markers and glucose metabolism status, extend the results obtained in vitro. Given that ferritin is inversely correlated with sTfR we expected a negative relationship between sTfR uncarboxylated osteocalcin rather than the negative observed in our study. However, our results are in agreement to those reported by Rajpathak and coworkers who observed, in a nested case-control study conducted in the framework of the Diabetes Prevention Program, a positive association between serum sTfR levels and T2DM risk even after adjusting by ferritin levels, thus suggesting another potential mechanism linking sTfR and T2DM unrelated to iron overload [7]. Increased levels of sTfR have been observed in vitro after insulin administration to rats (32) and it cannot be discard sTfR levels as a biomarker of other factors causally related to T2DM [7]. In our study subjects, sTfR levels could be the result of a compensatory mechanism for a reduction of free iron levels secondary to inflammatory or oxidative status common in old subjects at cardiovascular risk. Moreover, in addition to other epidemiological studies, we also showed positive associations of ferritin and sTfR with fasting glucose, insulin and HOMA-IR, but not with HOMA-BCF, suggesting that the contribution of iron metabolism to type 2 diabetes mellitus is basically related to the induction of insulin resistance more than through an effect on beta-cell function [3], [15]. In contrast to previous prospective studies suggesting that lower ratios of sTfR: ferritin were associated with increased risk of type 2 diabetes [2], [4], no impact on the association of sTfR:ferritin and osteocalcin or adiponectin was observed in our study. However, as expected, we observed a negative tendency in the association between this ratio and HOMA-IR.

Iron stores could also induce insulin resistance through induction of oxidative stress [39] and serum ferritin has been associated with circulating oxidized LDL lipoproteins and advanced oxidation products [40]. Nevertheless, we observed a significant relationship of iron markers and osteocalcin or adiponectin independently of oxidized LDL, suggesting that the associations between iron and insulin resistance could be additionally mediated by other pathways rather than oxidative stress.

One concern of the present study is that ferritin concentrations may reflect other physiological aspects rather than iron storage, especially subclinical systemic inflammation related to insulin resistance. This is more relevant in our subjects because they are old, obese and at high cardiovascular risk. The cross-sectional nature of our assessment also hinders the possibility of a proper ascertainment of the direction of the causal sequence and we acknowledge this limitation. Also, because the associations between iron metabolism and osteocalcin forms observed in our study are weak, we must be cautious to consider iron metabolism markers as predictors of osteocalcin levels. Therefore, further prospective investigations should be designed to confirm these associations.

We tried to minimize the potential confounding by obesity and inflammation controlling for BMI and oxidative and inflammatory status (CRP, adiponectin, oxLDL) in the multivariate models. However, we cannot rule out the possibility that our results may have been influenced by other unmeasured factors. In any case, the high cardiovascular risk of our study subjects is to our advantage because they are a homogeneous population with smaller between-subjects variability in cardiovascular risk factors than a sample of the general population.

In sum, we described an inverse association between serum ferritin and sTfR with total or uncarboxylated osteocalcin and adiponectin in subjects at high cardiovascular risk. These findings suggest that body iron metabolism may contribute to the induction of insulin resistance through the inhibition of adiponectin and osteocalcin thus providing support for the hypothesis that iron metabolism could contribute to the origin of type 2 diabetes mellitus. Further research is warranted to understand the exactly mechanisms by which ferritin and sTfR levels induce insulin resistance.
